# A *Caenorhabditis elegans* ortholog of human selenium-binding protein 1 is a pro-aging factor protecting against selenite toxicity

**DOI:** 10.1016/j.redox.2019.101323

**Published:** 2019-09-11

**Authors:** Karl Köhnlein, Nadine Urban, David Guerrero-Gómez, Holger Steinbrenner, Pavel Urbánek, Josephine Priebs, Philipp Koch, Christoph Kaether, Antonio Miranda-Vizuete, Lars-Oliver Klotz

**Affiliations:** a Institute of Nutritional Sciences, Nutrigenomics Section, Friedrich-Schiller-Universität Jena, Jena, Germany; b Instituto de Biomedicina de Sevilla (IBIS), Hospital Universitario Virgen del Rocío/CSIC/Universidad de Sevilla, Sevilla, Spain; c Leibniz Institute on Aging - Fritz Lipmann Institute, Jena, Germany

**Keywords:** Selenium-binding protein, Stress signaling, *Caenorhabditis elegans*, Lifespan

## Abstract

Human selenium-binding protein 1 (SELENBP1) was originally identified as a protein binding selenium, most likely as selenite. SELENBP1 is associated with cellular redox and thiol homeostasis in several respects, including its established role as a methanethiol oxidase that is involved in degradation of methanethiol, a methionine catabolite, generating hydrogen sulfide (H_2_S) and hydrogen peroxide (H_2_O_2_). As both H_2_S and reactive oxygen species (such as H_2_O_2_) are major regulators of *Caenorhabditis elegans* lifespan and stress resistance, we hypothesized that a SELENBP1 ortholog in *C. elegans* would likely be involved in regulating these aspects.

Here we characterize Y37A1B.5, a putative selenium-binding protein 1 ortholog in *C. elegans* with 52% primary structure identity to human SELENBP1. While conferring resistance to toxic concentrations of selenite, Y37A1B.5 also attenuates resistance to oxidative stress and lowers *C. elegans* lifespan: knockdown of Y37A1B.5 using RNA interference resulted in an approx. 10% increase of *C. elegans* lifespan and an enhanced resistance against the redox cycler paraquat, as well as enhanced motility. Analyses of transgenic reporter strains suggest hypodermal expression and cytoplasmic localization of Y37A1B.5, whose expression decreases with worm age. We identify the transcriptional coregulator MDT-15 and transcription factor EGL-27 as regulators of Y37A1B.5 levels and show that the lifespan extending effect elicited by downregulation of Y37A1B.5 is independent of known MDT-15 interacting factors, such as DAF-16 and NHR-49. In summary, Y37A1B.5 is an ortholog of SELENBP1 that shortens *C. elegans* lifespan and lowers resistance against oxidative stress, while allowing for a better survival under toxic selenite concentrations.

## Highlights

• Y37A1B.5 is a *C. elegans* ortholog of human selenium binding protein 1 (SELENBP-1).
• Y37A1B.5 is a cytoplasmic protein predominantly expressed in hypodermal cells.
• *Y37A1B.5* knockdown by RNAi causes *C. elegans* (mean and maximum) lifespan extension.
• Y37A1B.5 enhances selenite resistance but sensitizes towards oxidative stress.
• Expression of *Y37A1B.5* is modulated by MDT-15 and EGL-27.

## Graphical abstract

**Figure:**
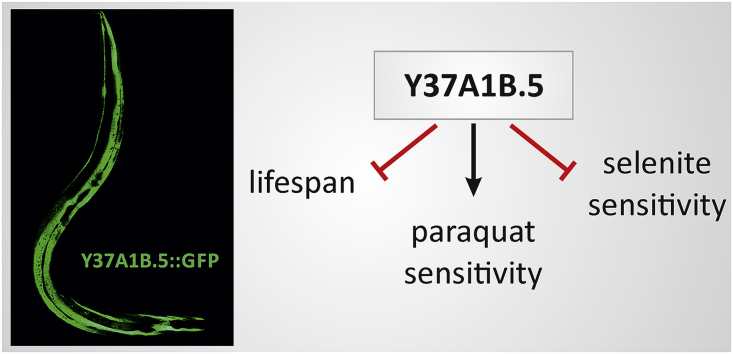

## 1 Introduction

Selenium (Se) is an essential trace element for humans and animals that is biologically active mostly in the form of selenocysteine built into selenoproteins. The human selenoproteome consists of 25 selenoproteins and includes five glutathione peroxidase (GPx), one methionine sulfoxide reductase (Msr) and three thioredoxin reductase (TrxR) isoform(s), which are involved in the removal of reactive oxygen species (ROS) such as hydroperoxides, the repair of oxidized methionine residues in proteins and the maintenance of cellular and systemic redox homeostasis [[1], [2], [3]]. Adequate Se intake has been associated with protection against oxidative stress-related adverse conditions and diseases such as cancer or inflammatory disorders in humans. On the other hand, Se oversupply causes toxicity, for example through redox cycling and Se compounds thus acting as “pro-oxidants” [[1], [2], [3]].

The nematode *Caenorhabditis elegans* is an interesting case with respect to selenoprotein and Se biochemistry: whereas the human genome encodes a total of 25 selenoproteins [4], *C. elegans* has only one selenoprotein, thioredoxin reductase-1 (TRXR1) [5,6]. Although *C. elegans* expresses a functional machinery allowing for incorporation of selenocysteine into this single protein, deletion of the *trxr-1* gene does not only not shorten *C. elegans* lifespan [7], but also does not affect sensitivity of *C. elegans* towards toxic effects of inorganic Se compounds on survival, growth and development [8,9]. While *trxr-1*-deficient nematodes contain considerably less Se compared to wild-type *C. elegans* when grown under basal conditions without Se supplementation, both strains incorporate Se with similar efficiency upon exposure to selenite [9]. Two further types of Se-containing proteins were described in mammalian cells: those that have non-specifically incorporated selenomethionine instead of methionine and the small group of selenium-binding proteins [10]. The most prominent of the latter is selenium-binding protein 1 (SELENBP1), originally identified as a protein binding Se, most likely as selenite [11]. Neither the mode of selenite binding nor the physiological significance of SELENBP1 have been fully elucidated. Nevertheless, some associations with (patho-)physiological processes were described, including a link to cell differentiation [12,13] and a correlation between low SELENBP1 expression levels in tumor tissue and poor clinical outcome (for review, see Ref. [14]).

Interestingly, SELENBP1 is associated with cellular redox and thiol homeostasis in several respects: (i) specific cysteine residues in SELENBP1 were proposed to mediate selenite binding or the interaction with metal ions [15,16]; (ii) SELENBP1 expression was found to be inversely related to activity and/or expression of the major antioxidant selenoenzyme GPx1 [14,17]; (iii) SELENBP1 was also shown to be involved in controlling the export of glutathione (GSH) from breast cancer cells and thereby affecting cellular resistance against oxidative stress [17]; (iv) lastly, a unique enzymatic activity of SELENBP1 was recently identified [18]. It is a methanethiol oxidase generating hydrogen sulfide (H_2_S) and hydrogen peroxide (H_2_O_2_) from a bacterial methionine degradation product, methyl mercaptan (methanethiol). Both H_2_S and ROS (such as H_2_O_2_) [19] as well as, more generally, redox processes and thiol metabolism [20] are major regulators of *C. elegans* lifespan and stress resistance. Therefore, we hypothesized that a SELENBP1 ortholog in *C. elegans* would likely be involved in regulating these aspects.

We here describe Y37A1B.5, a putative *C. elegans* ortholog of human SELENBP1, and define its role in regulating stress resistance and lifespan.

## 2 Materials and methods

### 2.1 Materials

Chemicals were purchased from Sigma-Aldrich (Munich, Germany) or Carl Roth (Karlsruhe, Germany), unless stated otherwise. Primers were obtained from Life Technologies (Darmstadt, Germany).

### 2.2 *C. elegans* maintenance and treatments

*C. elegans* strains were provided by the *Caenorhabditis* Genetics Center (CGC, University of Minnesota, USA), which is supported by the National Institutes of Health-Office of Research Infrastructure Programs. The following strains were used: wild-type Bristol N2; EU31, *skn-1(zu135) IV*; CF1038, *daf-16(mu86) I*; RB1716, *nhr-49(ok2165) I*. *E. coli* OP50 was also received from CGC. For knockdown experiments, RNAi clones were derived from the Ahringer library (Source BioScience, Nottingham, UK) [21] and grown using the appropriate antibiotics. Nematodes were grown, maintained and treated at 20°C on nematode growth medium (NGM) agar plates spotted with *E. coli* OP50 as food source, as described elsewhere [22]. For stress resistance assays, heat-inactivated bacteria (45 min at 65°C) were used.

Synchronized worm populations were used in all experiments. To this end, worms were washed off the plates, then the eggs collected and washed by centrifugation in sterile ultrapure H_2_O. Eggs were transferred to fresh NGM agar plates and allowed to hatch and grow for 64 h to young adulthood before being transferred to fresh plates for further experiments. For long-term incubations, nematodes were washed off the plates with S-basal buffer daily and were transferred to freshly prepared NGM agar plates to separate nematodes from progeny. Experiments were carried out with young adult worms, 64 h after synchronization, except for two experiments with exposure to RNAi from egg (see Table 2).

For RNAi experiments, 1 mM isopropyl-β-D-thiogalactoside (IPTG), 100 μg/ml ampicillin and, if necessary, 12.5 μg/ml tetracycline were added to NGM agar. On the evening before the experiment, agar plates were spotted with *E. coli* HT115 containing the L4440 empty vector (RNAi control) or the respective DNA fragment cloned into the L4440 vector and allowed to dry overnight.

For selenite toxicity assays, worms were treated with Y37A1B.5 RNAi starting at the egg stage for 72 h. Worms were then exposed to sodium selenite (0, 10, 20 or 30 mM) for 17 h in 150 μl of heat-inactivated bacteria suspension and shaken at 900 rpm on an orbital shaker. The assay was performed in triplicates in a 96-well format. After 17 h, viability was scored after transfer onto NGM plates.

### 2.3 Lifespan assays

Lifespan analyses of nematodes under RNAi conditions were performed using *E. coli* HT115 containing either L4440 empty vector (control) or the respective DNA fragment in L4440 starting at young adult stage. For the first 10 days, nematodes were transferred to fresh plates daily; thereafter, they were transferred every other day. Worms showing no movement, no reaction to gentle stimulation and no pharyngeal pumping were scored as dead. Worms lost or disintegrated, e.g. due to internal hatchings, were censored. Experiments were performed in multiple technical replicates (usually quintuplicates).

### 2.4 Stress resistance assays

Following RNAi or control treatment for five days, worms were exposed to the redox cycler paraquat. For paraquat exposure, worms were transferred to plates spotted with heat-inactivated bacteria and containing 300 mM paraquat; survival was scored hourly.

### 2.5 Assessment of locomotion, body area and body length

Nematodes were exposed to RNAi or control treatment for five, seven (body area, length) or ten (locomotion) days.

On day five or seven, 10 nematodes were transferred to S-basal with 0.1% Triton X-100. Worms were pipetted onto an objective slide and photographs of single worms were taken on an inverted Nikon Eclipse Ti-E fluorescence microscope. Each photograph was analyzed using the Nikon software NIS elements. Ten worms were analyzed for each treatment group, and the experiment was performed at least three times.

On day ten, single nematodes were transferred to S-basal medium containing 0.1% Triton X-100. Single worms were then pipetted, in a drop of 1 μl S-basal, onto an objective slide, and 10-s-movies were recorded under an inverted Nikon Eclipse Ti-E fluorescence microscope. For each treatment group, 10 worms were recorded, and the experiment was performed at least three times. Pathlength analysis was performed using Nikon tracking software (NIS elements).

### 2.6 Fecundity

64 h after synchronization, ten single nematodes of each treatment group were each transferred to one NGM RNAi agar plate to lay eggs. 24 h later the ten worms were transferred to ten new agar plates spotted with the respective bacteria and again allowed to lay eggs. The procedure was repeated until nematodes ceased to lay eggs. Each time, after transfer of worms, the remaining plates and eggs were placed back to the incubator. Worms (progeny) were allowed to hatch and grow until reaching the L3 larvae stage (48 h) and then counted. The experiment was performed three independent times.

### 2.7 Quantitative reverse transcription-PCR (qRT-PCR)

Worms were collected and shock-frozen in liquid nitrogen. Worms were lysed in TRIzol reagent (Thermo Scientific) and total RNA was isolated using acid guanidinium thiocyanate-phenol-chloroform extraction. RNA (1 μg) was reversely transcribed using GoScript Reverse Transcriptase (Promega) or RevertAid Reverse Transcriptase (Thermo Scientific), according to the manufacturers’ instructions, and subjected to qPCR analysis using SsoAdvanced Universal SYBR Green Supermix and a CFX Connect cycler (Bio-Rad Laboratories AG, Munich, Germany). Experiments were conducted in triplicates. *cdc-42*, *tba-1* or *pmp-3* were used as housekeeping genes for relative quantitation of mRNAs of interest [23]. Sequences of primers used for PCR are compiled in Table 1.

**Table 1:** Primer pairs used for qPCR analyses.

Gene name	Accession No. or Reference	Forward primer	Reverse primer
*Y37A1B.5*	NM_001268848.1	TTTTAGAATTCATCCTGTTGAGGAG	AAGAGCCCATCCACTTACTTTTT
*mdt-15*	NM_171134.6	ACGACAGCAGGAAACACTCC	TGATGGAGCAGGCAATCCTC
*egl-27*	NM_171011.3	CCATCAGGAAGAGCGTGTCA	CACGCTGGAACTTGAGATGG
*cdc-42*	[23]	AGCCATTCTGGCCGCTCTCG	GCAACCGCTTCTCGTTTGGC
*tba-1*	[23]	TCAACACTGCCATCGCCGCC	TCCAAGCGAGACCAGGCTTCAG
*pmp-3*	[23]	TGGCCGGATGATGGTGTCGC	ACGAACAATGCCAAAGGCCAGC

### 2.8 RNA sequencing (RNA-Seq)

Sequencing of RNA samples was done following their reverse transcription using Illumina's next-generation sequencing methodology [24]. Specifically, quality check and quantification of total RNA was done using the Agilent Bioanalyzer 2100 in combination with the RNA 6000 nano kit (Agilent Technologies). Approximately 1 μg of total RNA was used for library preparation using the TruSeq stranded mRNA library preparation kit (Illumina) following the manufacturer's description. Quantification and quality check of libraries was done using the Agilent Bioanalyzer 2100 in combination with the DNA 7500 kit. Libraries were sequenced on a HiSeq2500 running in 51cycle/single-end/high-output mode using sequencing chemistry v3. All libraries were pooled and sequenced in three lanes. Sequence information was extracted in FastQ format using Illumina's bcl2fastq v.1.8.4. Sequencing resulted in around 40 ± 4 million reads per sample.

### 2.9 RNA expression analysis

For expression analysis, the raw RNA-Seq reads were quality-trimmed and filtered for low complexity with the tool preprocess from the SGA assembler (version 0.10.13, parameters -q 30 -m 50 --dust) [25]. The passed reads were mapped to the *C. elegans* genome (WBcel235, release 90, http://aug2017.archive.ensembl.org/Caenorhabditis_elegans/Info/Annotation) with Ensembl gene annotation using TopHat2 (version 2.1.0; conservative settings, i. e --b2-sensitive --no-coverage-search --no-novel-juncs --no-novel-indels --transcriptome-max-hits = 1) [26]. Next, all reads mapping uniquely to an Ensembl gene were counted using FeatureCounts (version 1.5.0; multi-mapping or multi-overlapping reads were not counted, stranded mode was set to “–s 2”, Ensembl release 90 gene annotation) [27].

The table of raw counts per gene per sample was analyzed with R (version 3.4.1) using the package DESeq2 (version 1.16.1) [28] to test for differential expression. *Y37A1B.5* RNAi samples were contrasted with the control samples (L4440 empty vector). For each gene of the comparison, the p-value was calculated using the Wald significance test. Resulting p-values were adjusted for multiple testing with Benjamini & Hochberg correction. Genes with an adjusted p-value <0.05 are considered differentially expressed (DEGs). The log2-fold changes (LFC) were shrunken with lfcShrink to control for variance of LFC estimates for genes with low read counts.

### 2.10 Pathway enrichment

For the identification of enriched pathways, DEGs were entered in the GEne SeT AnaLysis Toolkit “WebGestalt” (www.webgestalt.org) as a ranked list, with the LFC representing the rank for each DEG. As a threshold for significantly enriched pathways, FDR was set to 0.05. We performed Gene Set Enrichment Analysis (GSEA) using the KEGG pathway database for *C. elegans*. The normalized enrichment score displays positively (black) and negatively (gray) related categories, which corresponds to up- or downregulated pathways, respectively.

### 2.11 Generation of transgenic strains

For the generation of a *Y37A1B.5* translational reporter construct, a region of 2988 bp upstream of the translation initiation codon ATG (promoter region) and the complete genomic coding sequence of the *Y37A1B.5* gene (excluding the translation termination codon) were amplified as a single PCR-fragment from the N2 wildtype worm genomic DNA, using primers with pre-designed BamHI and Cfr9I restriction enzyme sites (see Fig. 3A). The amplified region was digested using BamHI and Cfr9I and inserted into the pPD95_77 empty vector at the BamHI and XmaI sites. pPD95_77 was a gift from Andrew Fire (Addgene plasmid #1495; http://n2t.net/addgene:1495;RRID:Addgene_1495). The final construct *pY37A1B.5::Y37A1B.5::gfp* was injected into the N2 wildtype strain at 20 ng/μl along with the *rol-6(su1006)* coinjection marker at 50 ng/μl. Stable transmitting lines were used for image analysis.

### 2.12 Fluorescence microscopy

Adult nematodes were placed on microscope slides coated with 3% agarose, anaesthetized with 10 mM sodium azide, and covered with coverslips. GFP fluorescence was analyzed on a Nikon Eclipse Ti-E fluorescence microscope using appropriate filters (GFP: ex. 472±30 nm, em. 520±35 nm). Pictures to determine localization were taken on a Zeiss Axio Imager Z2 microscope. For quantitation of the GFP signal intensity, worms were marked as regions of interest (ROI) – either automatically using the binary threshold of the GFP signal or manually using the brightfield overlay image. Total GFP intensity and area of each ROI was then measured and GFP intensity was normalized to worm area and subtracted from background signal to obtain the relative GFP fluorescence intensity for each worm.

For analysis of GFP expression under stress, nematodes showing the roller phenotype were transferred to plates that were spotted with heat-inactivated bacteria containing the respective compound. 24 h later, GFP expression in those worms was analyzed as described above.

### 2.13 Statistical analysis

Data are expressed as means +SD unless stated otherwise. For lifespan analyses, statistical calculations were performed using JMP software (SAS Institute Inc., Cary, NC, USA), applying the log-rank test. Median and mean survival was calculated by log-rank test for each of the technical replicates in one experiment (i.e. biological replicate). Maximum lifespan in each technical replicate was defined as the last day on which a worm was scored as alive. Mean maximum lifespan for each biological replicate was then determined as the mean of all technical replicates. All other calculations were performed using GraphPad Prism (GraphPad Software, San Diego, California, USA). Statistical significances were calculated using one-way ANOVA (with Bonferroni post-test), Mann-Whitney test, or *t*-test where appropriate. The minimum level of significance was set to p < 0.05.

### 2.14 Data availability

The data discussed in this publication have been deposited in NCBI's Gene Expression Omnibus [29] and are accessible through GEO Series accession number GSE134196 (https://www.ncbi.nlm.nih.gov/geo/query/acc.cgi?acc=GSE134196).

## 3 Results and discussion

### 3.1 Y37A1B.5 *is a* C. elegans *ortholog of SELENBP1, affecting stress resistance and lifespan*

**Fig. 1:**
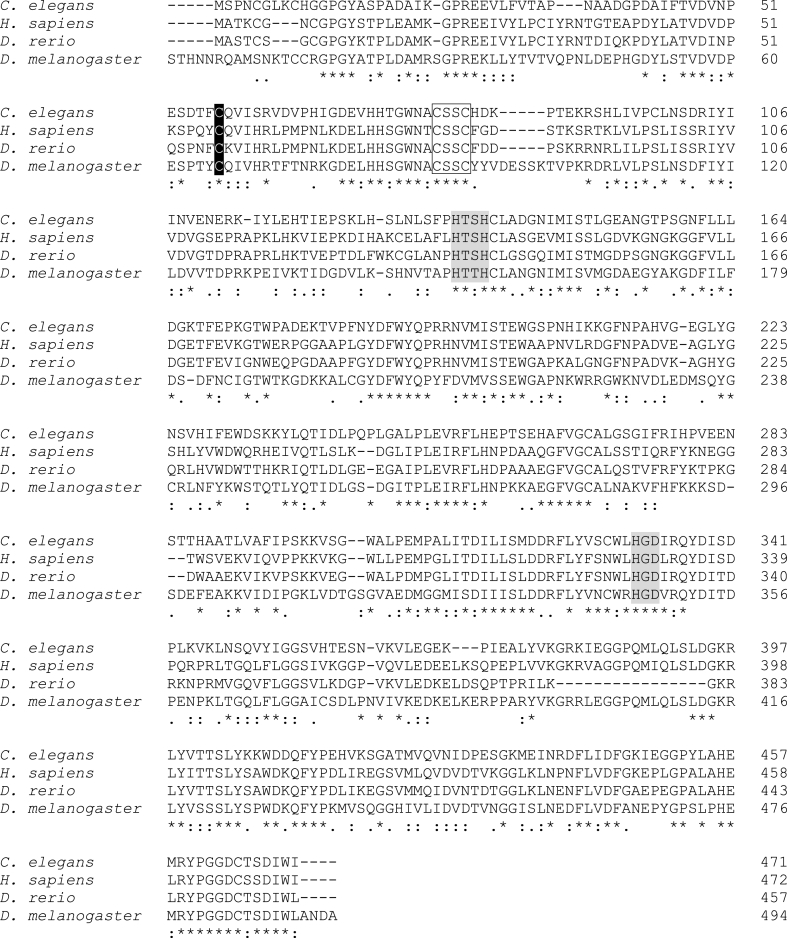
Y37A1B.5 displays high sequence homology with SELENBP1. Alignment of *C. elegans* Y37A1B.5 (NCBI Ref Seq#: NP_001255777.1) with human SELENBP1 (GenBank accession#: CAG33133.1) and orthologs from *Danio rerio* (NCBI Ref Seq#: NP_956864.1) and *Drosophila melanogaster* (LP16180p; GenBank accession#: AAV36940.1). The alignment identifies 52% identical aminoacyl residues for SELENBP1 and Y37A1B.5. Asterisk (*): fully conserved residue; colon (:): conservation between groups of strongly similar properties, Gonnet PAM 250 matrix > 0.5; period (.): conservation of weakly similar properties between groups, Gonnet PAM 250 matrix < 0.5. Multiple sequence alignment was performed using CLUSTAL O (1.2.4) [43]. Selected conserved motifs are highlighted as follows: Cys57, highlighted in black; thioredoxin motif (CxxC, transparent box); histidine-containing metal binding motifs (HxD, HxxH, gray background); see text for details.

We conducted a BLAST search of the *C. elegans* non-redundant protein sequence database for homologs of human SELENBP1 (GenBank accession# CAG33133). Y37A1B.5 is the *C. elegans* protein most similar in primary structure to SELENBP1, with 52% identity in amino acid sequence. Several conserved regions were identified by multiple sequence alignment of Y37A1B.5 with human SELENBP1 and its homologs in two other non-mammalian species, *Drosophila melanogaster* and *Danio rerio* (Fig. 1). One example is Cys57, corresponding to Cys57 in SELENBP1 (highlighted in black in Fig. 1). Cys57 was hypothesized to be involved in Se binding [15] and demonstrated to be a crucial determinant of stability and effects of overexpressed SELENBP1 in cultured cells [30]. Further conserved motifs include a thioredoxin motif [31,32] present at the active site of redoxin family proteins (CxxC, transparent box in Fig. 1) [33], and histidine-containing metal binding motifs (HxD, HxxH, gray background in Fig. 1) [32,34].

Next, we tested whether expression of *Y37A1B.5* may be altered in relation to worm age, and whether it affects *C. elegans* lifespan. For this purpose, we determined *Y37A1B.5* mRNA levels during the course of *C. elegans* adulthood, followed by analysis of the consequences of *Y37A1B.5* knockdown by RNA interference (RNAi).

**Fig. 2:**
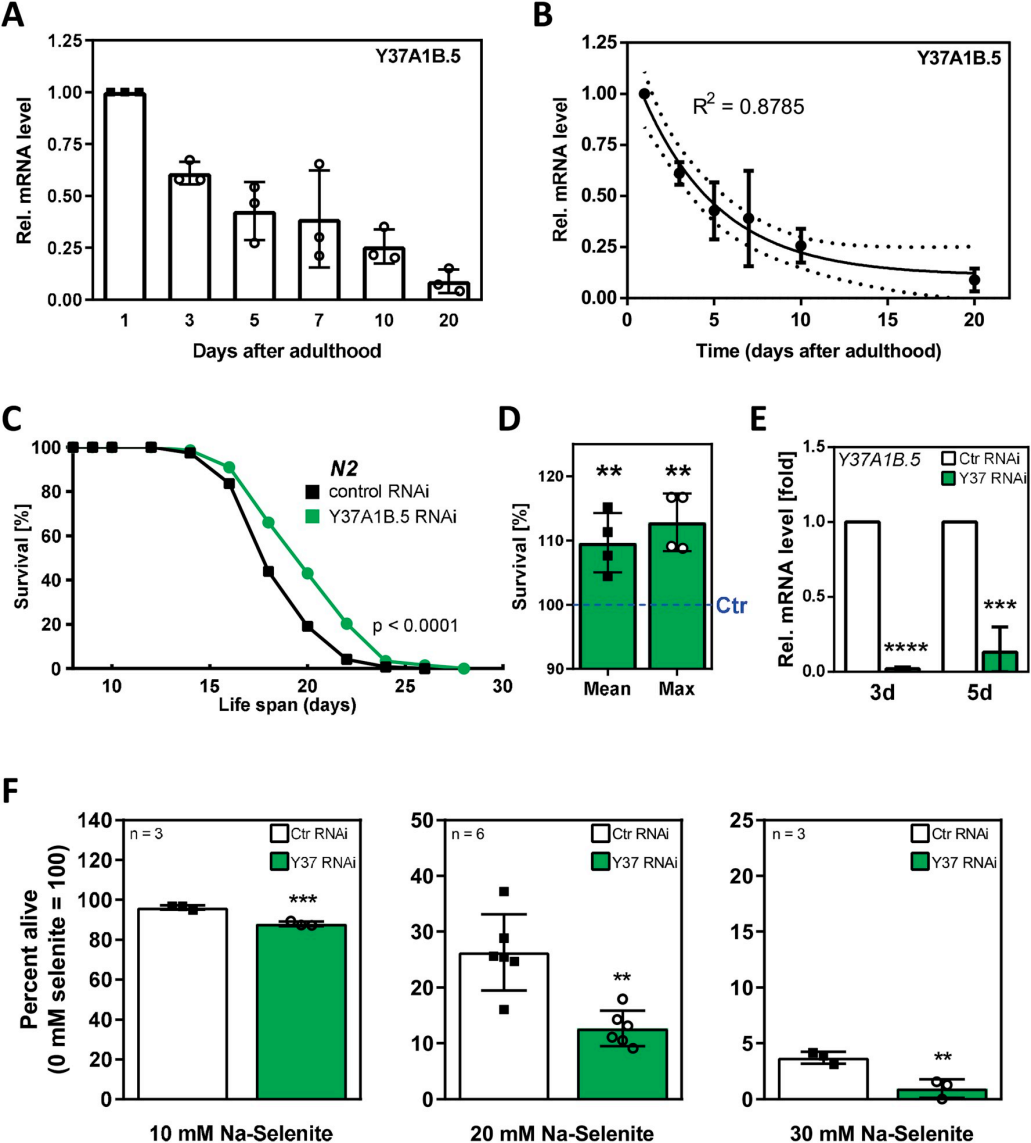
*Y37A1B.5* mRNA levels decrease with age and its downregulation increases lifespan. **(A)** Relative *Y37A1B.5* mRNA levels of N2 wild-type worms after 1, 3, 5, 7, 10 or 20 days, starting 64 h after synchronization, as determined in three independent experiments using qRT-PCR. Data were first normalized to mRNA levels of the housekeeping gene and then against the expression level of day 1. Data shown are relative means ± SD. **(B)** Exponential regression of data from graph (A). Data shown are means ± 95% confidence band. **(C)** Survival of nematodes depleted of *Y37A1B.5* through RNAi (p<0.0001 *vs.* control, log-rank test). Age-synchronized young adult worms were subjected to *Y37A1B.5*-specific or control RNAi. Survival was monitored daily until the end of the reproduction period and every second day thereafter. The lifespan experiment was conducted in quintuplicates and was performed four independent times (for details, see Table 2). One representative experiment is shown. **(D)** Relative mean and maximum lifespan of four independent lifespan experiments with *Y37A1B.5* RNAi or control. Data were normalized to mean or maximum lifespan of respective control experiment (**p<0.01 vs. control; unpaired *t*-test). **(E)** Relative *Y37A1B.5* mRNA levels after three or five days of *Y37A1B.5* RNAi or control, starting at the young adult stage, as determined in three independent experiments using qRT-PCR. Data shown are relative means +SD. (***p<0.001 vs. respective control; *t*-test) **(F)** Toxicity of different selenite concentrations to *C. elegans* following RNAi using control or *Y37A1B.5*-specific plasmids (***p<0.001, **p<0.01 *vs.* control; unpaired *t*-test).

*Y37A1B.5* gene expression exponentially declines over the course of *C. elegans* adulthood, i.e. from the first day of adulthood to 20 days post adulthood (Fig. 2 A, B). Depletion of *Y37A1B.5* through RNAi, i.e. imitation of the later-age situation with respect to *Y37A1B.5* expression, resulted in an enhanced lifespan (Fig. 2C), with a significant extension of both mean and maximum lifespan (Fig. 2D; see Table 2 for details). Using RNAi, we achieved *Y37A1B.5* knockdown to yield mRNA levels of approx. 10 % of control (2 and 13% on days 3 and 5, respectively; Fig. 2E).

**Table 2:** Statistics for RNAi knockdown of *Y37A1B.5*^a^

Exp. No.	Strain, Treatment	Effect on Life Span	P (vs. Ctrl)^b^	Median Life Span (Days)	Mean Life Span (Days ± SEM)^c^	Mean Life Span (%)	Max Life Span (Days ± SEM)^c^	Max Life Span (%)	No. of Uncen-sored Worms	Total Number of Worms
Knockdown of *Y37A1B.5* (starting at young adult stage)										
**1** (see Fig. 2C)	N2 /L4440			18	19.0 ± 0.1	100	22.0 ± 0.6	100	184	394
	N2 /Y37A1B.5	↑	****	20	20.5 ± 0.2	107.7	24.0 ± 0.8	109.1	218	382
**2**	N2 /L4440			20	20.0 ± 0.1	100	24.0 ± 0.6	100	332	400
	N2 /Y37A1B.5	↑	****	22	22.2 ± 0.1	111.3	28.0 ± 1.0	116.7	359	400
**3**	N2 /L4440			20	19.4 ± 0.1	100	22.8 ± 0.4	100	284	400
	N2 /Y37A1B.5	↑	****	20	20.2 ± 0.2	104.4	24.8 ± 0.7	108.8	320	400
**4**	N2 /L4440			18	18.9 ± 0.2	100	24.0 ± 0.8	100	283	400
	N2 /Y37A1B.5	↑	****	22	21.8 ± 0.3	115.2	28.0 ± 1.0	116.7	365	400
Knockdown of *Y37A1B.5* (starting from egg)										
**1**	N2 /L4440			20	20.3 ± 0.2	100	24.4 ± 1.0	100	249	400
	N2 /Y37A1B.5	=	n.s.	20	20.4 ± 0.3	100.9	26.4 ± 1.0	108.2	335	400
**2**	N2 /L4440			18	17.9 ± 0.3	100	21.6 ± 0.4	100	324	400
	N2 /Y37A1B.5	↑	****	18	18.8 ± 0.1	104.9	25.2 ± 0.4	116.7	362	400

a Survival curves for all experiments are provided in Supplemental Figs. S1 and S2.

b Control: N2 /L4440; ****P<0.0001; n. s., not significant.

c 5 technical replicates.

These data suggest that, whereas *Y37A1B.5* expression may be dispensable for *C. elegans* at a later age, it appears to be required in early life, as knockdown of *Y37A1B.5* from egg does not lead to an unambiguous (reproducible) extension of lifespan, although no overt adverse effects of deficiency were noticed either (Table 2). The data also suggest that expression of *Y37A1B.5* comes at a cost, as knocking down *Y37A1B.5* in young adults causes an extension of lifespan.

We therefore sought for potential beneficial effects of Y37A1B.5. As SELENBP1 was originally detected as a protein binding Se in mice injected with high doses of selenite [11], we tested for the role of Y37A1B.5 in *C. elegans* exposed to 10, 20 or 30 mM selenite, which was shown before to be a concentration range toxic to the nematodes [9]. As demonstrated in Fig. 2F, *C. elegans* is significantly more sensitive to selenite at all tested concentrations upon knockdown of *Y37A1B.5* by RNAi, suggesting a protective role of the protein against acutely toxic Se exposure.

In summary, Y37A1B.5 is an ortholog of human SELENBP1 that protects *C. elegans* against selenite toxicity. Conversely, its downregulation enhances *C. elegans* lifespan.

### 3.2 Y37A1B.5 is a cytoplasmic protein predominantly expressed in hypodermal cells

**Fig. 3:**
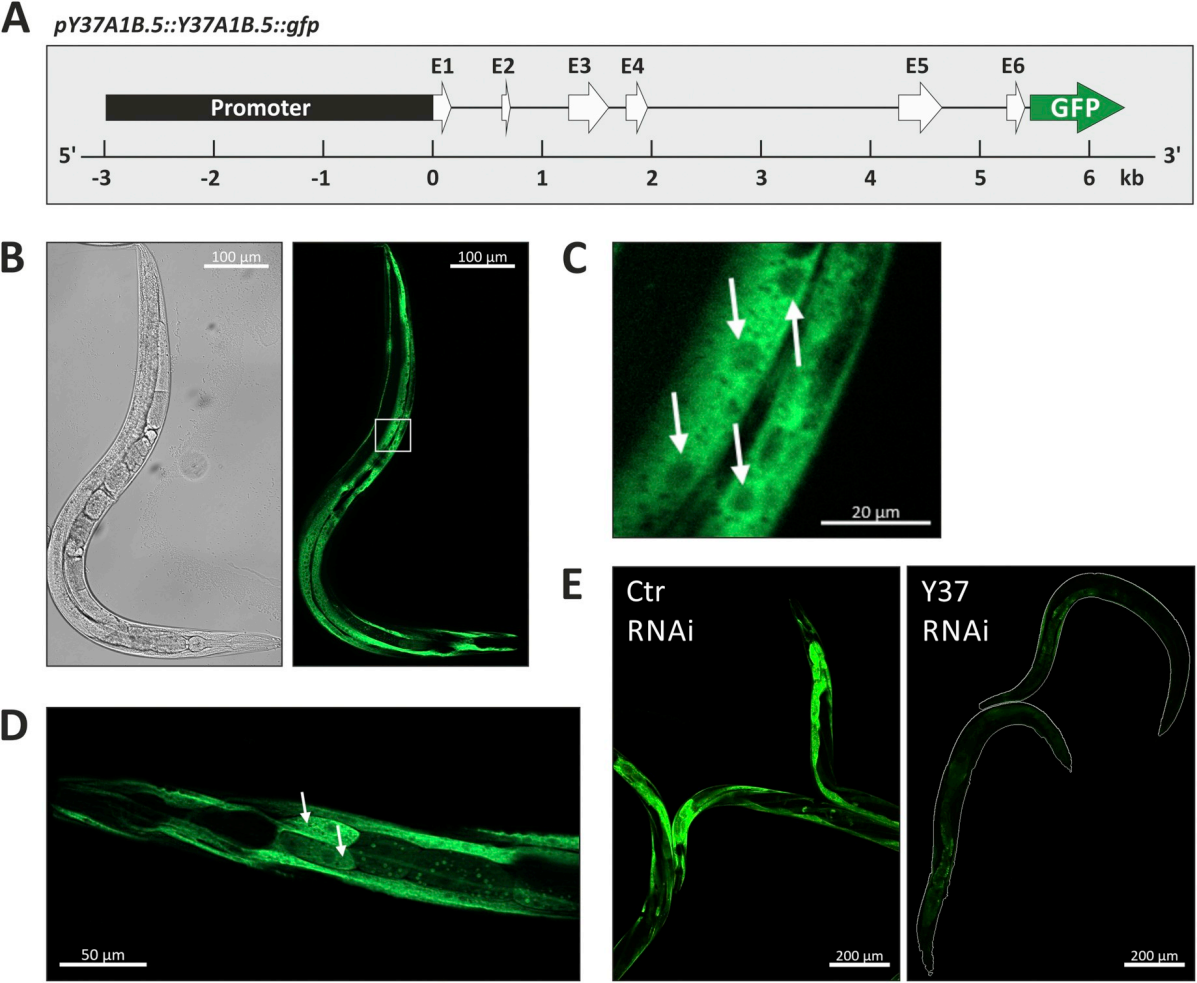
Y37A1B.5 is a cytoplasmic protein expressed in hypodermal cells. **(A)** Schematic representation of the translational reporter construct used in this study, *pY37A1B.5*::*Y37A1B.5*::*gfp*. (E1-E6, exons 1-6) **(B)** Hypodermal localization of Y37A1B.5::GFP. White box indicates region enlarged for (C). **(C)** Subcellularly, Y37A1B.5::GFP is localized primarily in the cytoplasm. White arrows indicate nuclei devoid of signal. **(D)** Few worms display fluorescence in anterior (arrows) or posterior (not shown) intestinal cells. **(E)** Comparison of RNAi using control (Ctr) or *Y37A1B.5*-specific (Y37) plasmids on Y37A1B.5::GFP reporter strain. White lines indicate worm shape.

We then set out to further characterize the expression pattern of *Y37A1B.5* and the physiological consequences thereof in the absence of added Se. To this end, we generated a translational fusion construct harboring 3 kb of *Y37A1B.5* endogenous promoter region plus the complete ORF fused to GFP (*pY37A1B.5::Y37A1B.5::gfp*), as depicted in Fig. 3A. Microscopic analysis of adult transgenic worms shows that GFP fluorescence is most prominent in hypodermal cells (Fig. 3B). Subcellularly, Y37A1B.5:GFP is found predominantly in the cytoplasm (Fig. 3C), which is true also for human SELENBP1 [12]. Sporadically, in few worms only, GFP fluorescence is also detected in some anterior or posterior intestinal cells (Fig. 3D).

As demonstrated in Fig. 3E, the RNAi approach chosen to analyze the effect of Y37A1B.5 on lifespan (Fig. 2) also effectively downregulated expression of the transgenic fusion protein as indicated by attenuated overall Y37A1B.5::GFP fluorescence.

### 3.3 Depletion of Y37A1B.5 enhances stress resistance and improves physiological parameters

**Fig. 4:**
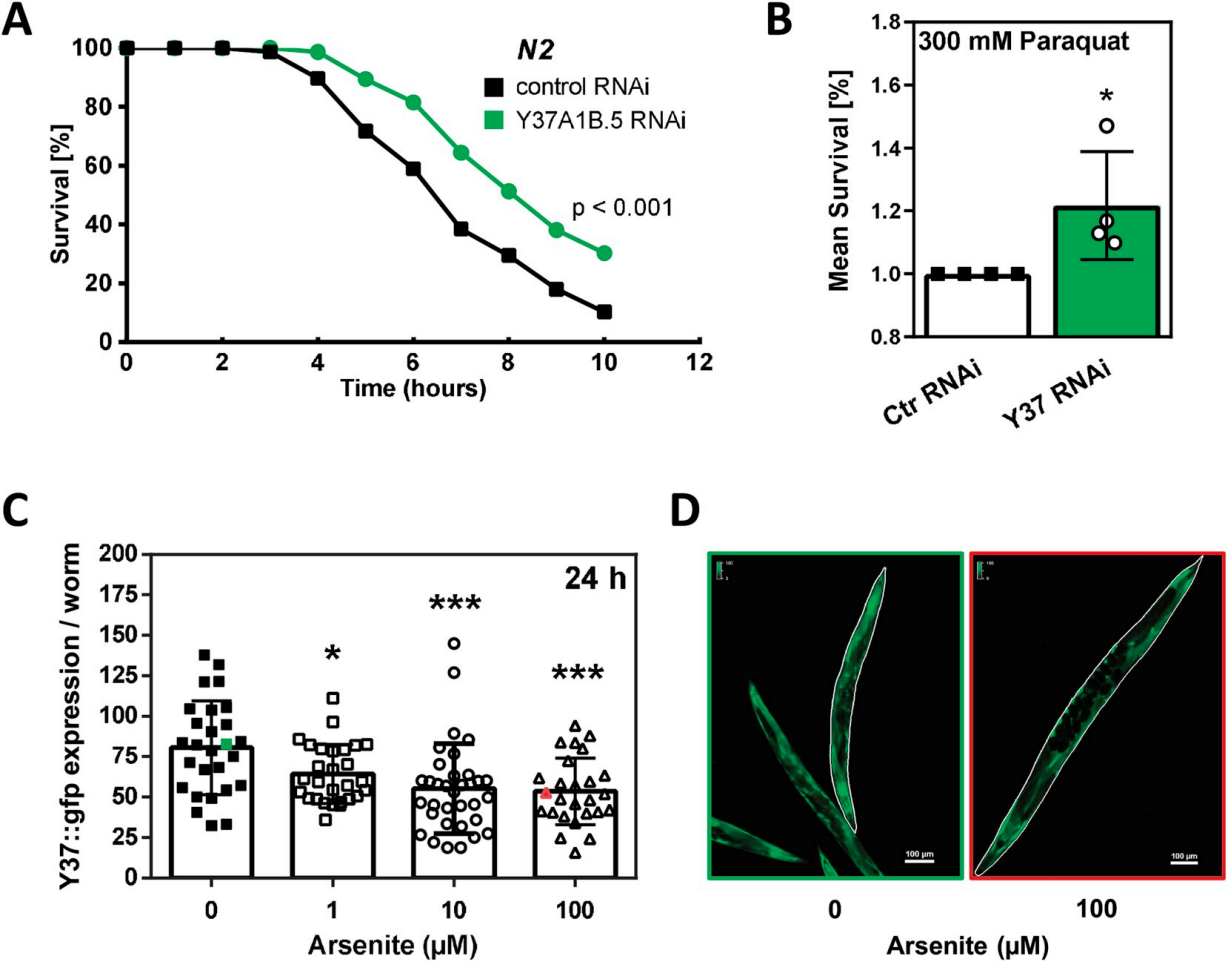
Y37A1B.5 is involved in the stress response of *C. elegans*. **(A)** N2 wildtype worms (young adult; 64 h after synchronization) were fed with *Y37A1B.5* RNAi or control bacteria for 144 h and then exposed to 300 mM of paraquat for 10 h. Survival was scored every hour. (p *vs.* control, log-rank test). **(B)** Relative mean survival of *Y37A1B.5* RNAi or control worms on 300 mM paraquat resulting from four independent experiments. Data were normalized to mean survival of control (*p<0.05 vs. control, Mann-Whitney test). **(C)** Y37A1B.5::GFP signals in young adult worms (64 h after synchronization) that were exposed to arsenite for 24 h. Each data point represents Y37A1B.5::GFP signal/worm in arbitrary units for one individual worm (*p<0.05, ***p<0.001; unpaired *t*-test). **(D)** Examples of worms evaluated in Fig. 4C (green, red data points), representing worms exposed to 0 μM (green box) and 100 μM (red box) arsenite.

In order to test whether the extended lifespan of worms following *Y37A1B.5* downregulation was accompanied by a change in resistance to stressful stimuli other than selenite, we exposed the nematodes to a lethal dose (300 mM) of the redox cycler paraquat. Surprisingly, and in contrast to the selenite exposure, after RNAi treatment for six days, *Y37A1B.5*-deficient worms exhibited a significantly better survival under paraquat exposure than worms fed with control RNAi (Fig. 4A and B; for details, see Table 3).

**Table 3:** Statistics for stress resistance analysis against paraquat after *Y37A1B.5* RNAi.

Exp. No.	Strain, Treatment	Effect on Survival	P (vs. Ctrl)^a^	Median Survial (h)	Mean Survival (h ± SEM)	Mean Survival (%)	No. of Uncensored Worms	Total No. of Worms
**1**	N2 /L4440			5	5.6 ± 0.2	100	69	80
	N2 /Y37A1B.5	=	p = 0.0556	6	6.2 ±0.1	109.9	38	49
**2**	N2 /L4440			4	5.5 ± 0.2	100	66	79
	N2 /Y37A1B.5	↑	****	9	8.0 ± 0.2	146.9	44	81
**3** (**see**Fig. 4A)	N2 /L4440			7	7.1 ± 0.2	100	70	78
	N2 /Y37A1B.5	↑	***	9	8.2 ± 0.2	116.8	53	76
**4**	N2 /L4440			8	7.8 ± 0.2	100	81	90
	N2 /Y37A1B.5	↑	****	10	8.8 ± 0.2	112.8	50	85

a Controls: N2 /L4440; ***P<0.001 ****P<0.0001; n. s., not significant.

Exposure of *pY37A1B.5::Y37A1B.5::gfp* transgenic worms to another stressful stimulus, arsenite, elicited an attenuation of Y37A1B.5::GFP expression (Fig. 4C and D), suggesting that downregulation of Y37A1B.5 may be part of a general *C. elegans* stress response.

**Fig. 5:**
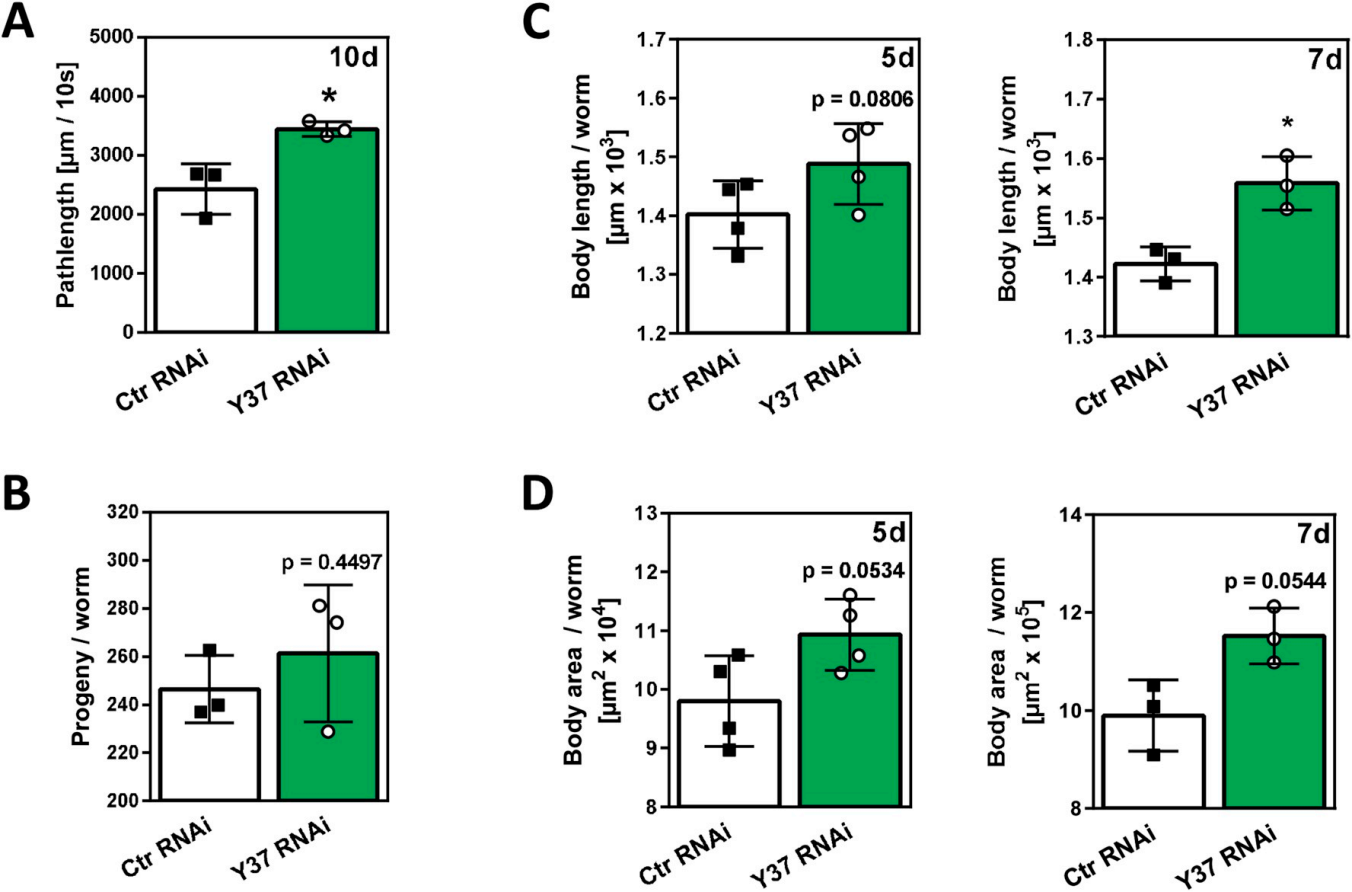
*Y37A1B.5* downregulation improves *C. elegans* health parameters. (**A)** Motility of worms after 10 days of RNAi treatment (*Y37A1B.5* RNAi *vs*. control); movements of individual worms were captured by a camera and distances covered per 10 s calculated using a tracking software. For an individual experiment, 10 worms were analyzed per group; three independent experiments were performed (*p<0.05, paired *t*-test). **(B)** Progeny per worm depleted of *Y37A1B.5* through RNAi. 64 h after synchronization worms were transferred to RNAi plates. Each day, single worms were transferred to a new agar plate until nematodes ceased to lay eggs. Worms were allowed to hatch from the eggs and to grow until reaching the L3 larval stage before being counted. The experiment was conducted in quintuplicates and performed three independent times (p>0.05 vs. control; paired *t*-test). **(C)** Body length [μm] per worm (*p<0.05, paired *t*-test) and **(D)** body area [μm^2^] per worm after *Y37A1B.5* RNAi. Young adult worms were exposed to control or *Y37A1B.5*-specific RNAi for 5 or 7 days. Pictures were taken and worms were measured using NIS elements software (Nikon). 10 worms were analyzed for each treatment group, and the experiment was repeated twice (7d) or three times (5d). Data shown are means ± SD (statistical analysis: paired *t*-test).

In line with their elevated stress resistance to paraquat and extended lifespan, the *Y37A1B.5*-deficient worms generally appeared to be healthier than their wild-type counterparts, particularly at older age. This was indicated also by the improved *C. elegans* motility upon knockdown of *Y37A1B.5* (Fig. 5A). No impairment of *C. elegans* fecundity was observed under these conditions (Fig. 5B), and there was a trend towards an increased body area and length, particularly at later stages (Fig. 5C and D).

### 3.4 Analysis of Y37A1B.5 knockdown-induced transcriptome changes

**Fig. 6:**
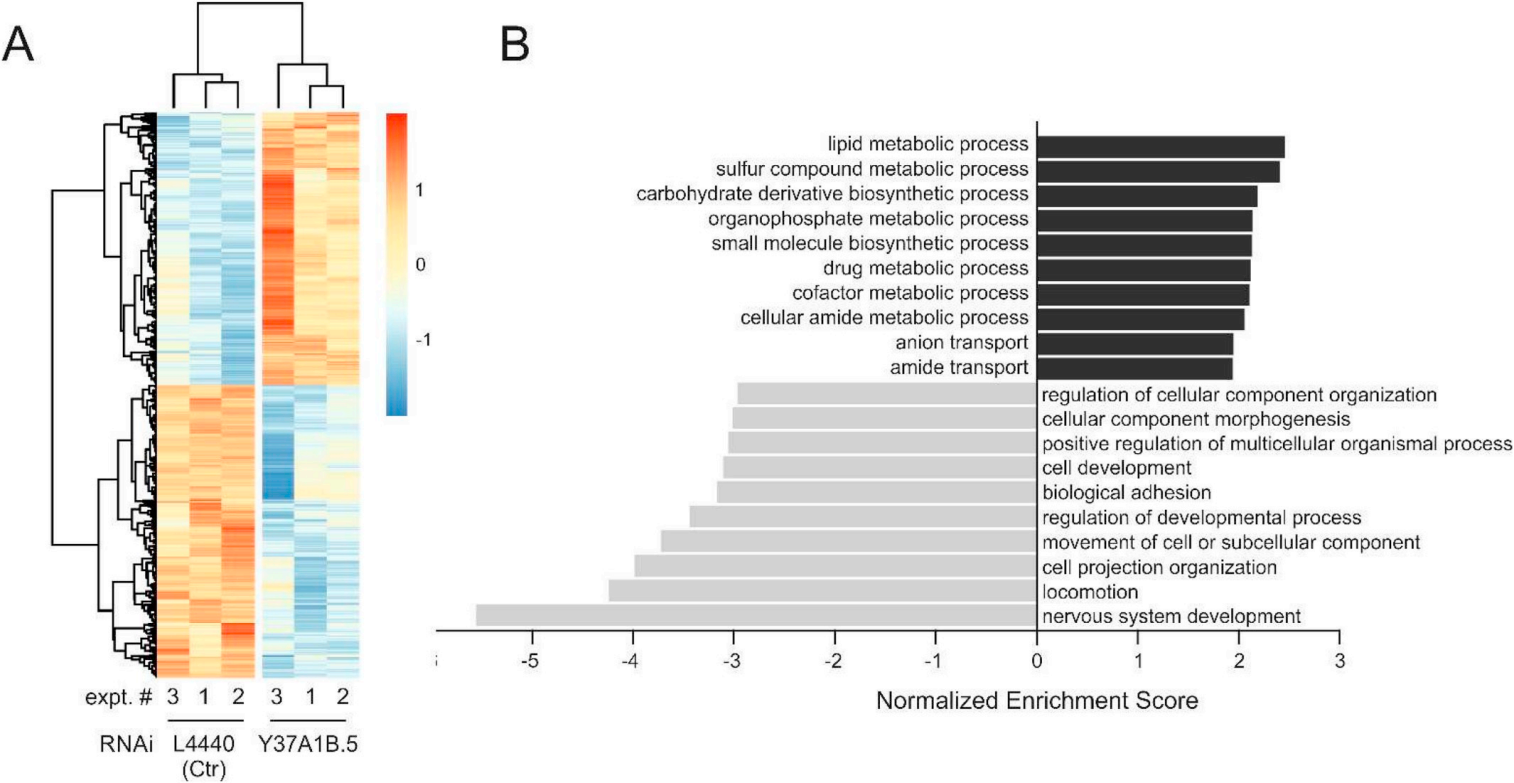
RNA-Seq analysis of transcriptome changes induced by *Y37A1B.5* knockdown. (A) Heat map of individual expression values of all 2474 differentially expressed genes (adjusted p-value < 0.05). Prior to heat map calculation, raw read counts were size-factor normalized (DESeq2, v. 1.16.1). The color scale represents row z-score. Hierarchical clustering of the samples or DEGs is represented by dendrograms at the top and left, respectively. (B) GSEA analysis for GO biological processes using WebGestalt 2019, with fold changes of differentially expressed genes indicated as rank. Analysis was performed using standard settings. Black (positive) or gray (negative) bars show “up-” or “downregulated” GO terms according to normalized enrichment scores.

In order to investigate the role of Y37A1B.5 in *C. elegans* metabolism, we performed gene expression analysis using RNA-Seq. RNA was isolated from *C. elegans* fed with bacteria expressing the empty RNAi vector L4440 or a *Y37A1B.5*-specific RNAi plasmid. Three independent experiments were performed (biological replicates), followed by reverse transcription of the RNA and Illumina Sequencing. Analysis for differentially expressed genes (DEGs, Supplemental Table 1) identified 2474 DEGs that are regulated consistently over all three replicates (Fig. 6A).

Clustering of DEGs according to Gene Ontology (GO) terms (Fig. 6B) revealed that Y37A1B.5 is involved in the regulation of metabolic processes, with sulfur compound metabolism and lipid/carbohydrate metabolic processes in prominent positions among the relevant GO terms.

The involvement of Y37A1B.5 in sulfur metabolic processes is expected for an ortholog of SELENBP1 (see Introduction). Its role in lipid and carbohydrate metabolic processes is in line with findings on the link between Selenbp1 with glucose/lipid metabolism in mice [35].

### 3.5 Y37A1B.5 knockdown-induced lifespan extension is independent of major stress-responsive transcription factors

**Fig. 7:**
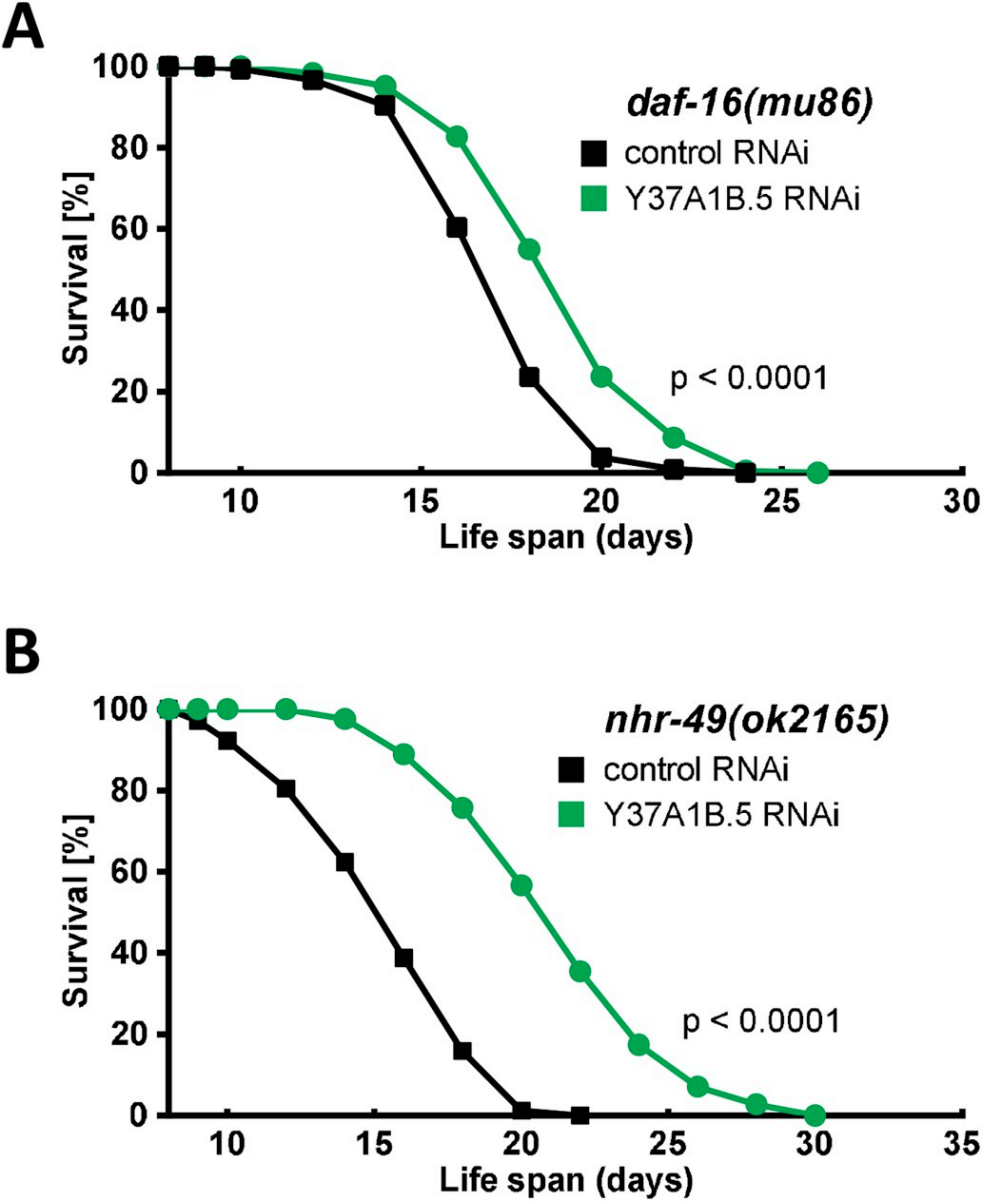
Lifespan extension elicited by depletion of *Y37A1B.5* is independent of transcription factors DAF-16 and NHR-49. Age-synchronized nematodes were subjected to *Y37A1B.5*-specific RNAi or control throughout their lifespan, starting 64  h after synchronization. Survival rates of **(A)***daf-16*(*mu86*) and **(B)***nhr-49*(*ok2165*) nematodes were monitored daily for survival until the end of the reproduction period and every second day thereafter. P values were determined by log-rank test. Representative survival curves are depicted (for details, see Table 4).

DAF-16 (the *C. elegans* ortholog of mammalian FOXO proteins) and SKN-1 (the Nrf2 ortholog) are among the most prominent transcription factors that are known to be involved in the regulation of fuel (glucose/lipid) metabolism and stress response [36,37]. Both contribute to *C. elegans* resistance under various stress conditions, including exposure to thiol-depleting agents [20]. Lifespan of DAF-16-deficient worms, i.e. *daf-16(mu86)* mutants (see Fig. 7A, Table 4), is lower than that of wildtype *C. elegans*, and *Y37A1B.5* knockdown extends lifespan to an extent similar to *Y37A1B.5* knockdown in wildtype worms (Table 2, Table 4). A similar effect is observed in our preliminary studies with SKN-1-deficient worms [*skn-1(zu135)*] (n = 1, >200 worms; Table 4, Fig. S4). Unexpectedly, therefore, neither DAF-16 nor SKN-1 appear to be involved in the lifespan extension elicited by *Y37A1B.5* knockdown (Fig. 7A, Table 4). Another major regulator of lipid metabolism, NHR-49, a transcription factor analogous to mammalian hepatocyte nuclear factor 4 (HNF4) and peroxisome proliferator-activated receptor α (PPARα), was demonstrated to also be involved in the *C. elegans* response to oxidative stress [38]. In order to test for a requirement of NHR-49 for lifespan extension by *Y37A1B.5* knockdown we used NHR-49-deficient worms, *nhr-49(ok2165)*. Lifespan of these worms is significantly lower than that of wildtype *C. elegans* (not shown), as previously described. Knockdown of *Y37A1B.*5 by RNAi, however, strongly enhances mean and maximum lifespan of NHR-49-deficient worms (Fig. 7B) by 27 to 36% and 34 to 43%, respectively (Table 4), i.e. much higher than in *daf-16(mu86)* and *skn-1(zu135)* mutants. This suggests that NHR-49, although not contributing to lifespan extension elicited by *Y37A1B.5* knockdown, antagonizes the lifespan extending effect of *Y37A1B.5* downregulation. Taken together, the transcription factors DAF-16 and NHR-49, and likely also SKN-1, are not involved in the lifespan extension mediated by *Y37A1B.5* knockdown.

**Table 4:** Statistics for RNAi knockdown of *Y37A1B.5* in knockout mutants^a^.

Exp. No.	Strain, Treatment	Effect on Life Span	P (vs. Ctrl)^b^	Median Life Span (Days)	Mean Life Span (Days ± SEM)^c^	Mean Life Span (%)	Max Life Span (days ± SEM)c	Max Life Span (%)	No. of Uncen-sored Worms	Total Number of Worms
Knockdown of *Y37A1B.5* in *daf-16(mu86)*										
1 (see Fig. 7A)	*daf-16* /L4440			18	17.6 ± 0.0	100	20.8 ± 0.4	100	311	400
	*daf-16* /Y37A1B.5	↑	****	20	19.3 ± 0.1	109.9	22.4 ± 0.4	107.7	370	400
**2**	*daf-16* /L4440			18	18.9 ± 0.1	100	22.0 ± 0.0	100	225	450
	*daf-16* /Y37A1B.5	↑	****	20	19.8 ± 0.2	104.6	24.0 ± 0.8	109.1	259	450
Knockdown of *Y37A1B.5* in *skn-1(zu135)*										
**1**	*skn-1* /L4440			17	17.4 ± 0.1	100	19.8 ± 0.4	100	210	231
	*skn-1* /Y37A1B.5	↑	****	19	19.2 ± 0.2	110	25.8 ± 0.7	130.3	204	230
Knockdown of *Y37A1B.5* in *nhr-49(ok2165)*										
**1**	*nhr-49* /L4440			16	15.8 ± 0.2	100	20.0 ± 0.0	100	248	400
	*nhr-49* /Y37A1B.5	↑	****	20	20.1 ± 0.3	127.4	26.4 ± 0.7	132.0	283	400
**2** (see Fig. 7B)	*nhr-49* /L4440			16	15.9 ± 0.1	100	19.2 ± 0.4	100	321	400
	*nhr-49* /Y37A1B.5	↑	****	22	21.6 ± 0.1	136.4	28.0 ± 0.0	145.8	295	400
**3**	*nhr-49* /L4440			16	15.4 ± 0.3	100	20.0 ± 0.0	100	298	316
	*nhr-49* /Y37A1B.5	↑	****	20	20.9 ± 0.2	135.3	27.5 ± 0.4	137.5	300	320

a Survival curves for all experiments are provided in Supplemental Figs. Figs. S3–S5.

b Controls: *daf-16* /L4440; *skn-1* /L4440; *nhr-49* /L4440; ****P<0.0001.

c 4-5 technical replicates.

### 3.6 Expression of Y37A1B.5 is modulated by transcriptional regulators MDT-15 and EGL-27

In order to identify transcriptional regulators of *Y37A1B.5* expression, we focused on two factors that are known to be involved in regulating *C. elegans* stress resistance and lifespan, and, in part, affect lipid metabolism, MDT-15 and EGL-27 [39,40]. MDT-15 is a transcriptional coregulator known to be involved in the regulation of lipid metabolism [39] and required for the *C. elegans* transcriptional response to (oxidative) stress as elicited by exposure to paraquat or arsenite [41]. EGL-27 is a transcriptional regulator that was shown to confer stress resistance, extending lifespan of *C. elegans* [40].

Using worms overexpressing *pY37A1B.5::Y37A1B.5::gfp* (Fig. 3), we tested for an effect of knocking down *mdt-15* or *egl-27* expression on *Y37A1B.5* expression.

**Fig. 8:**
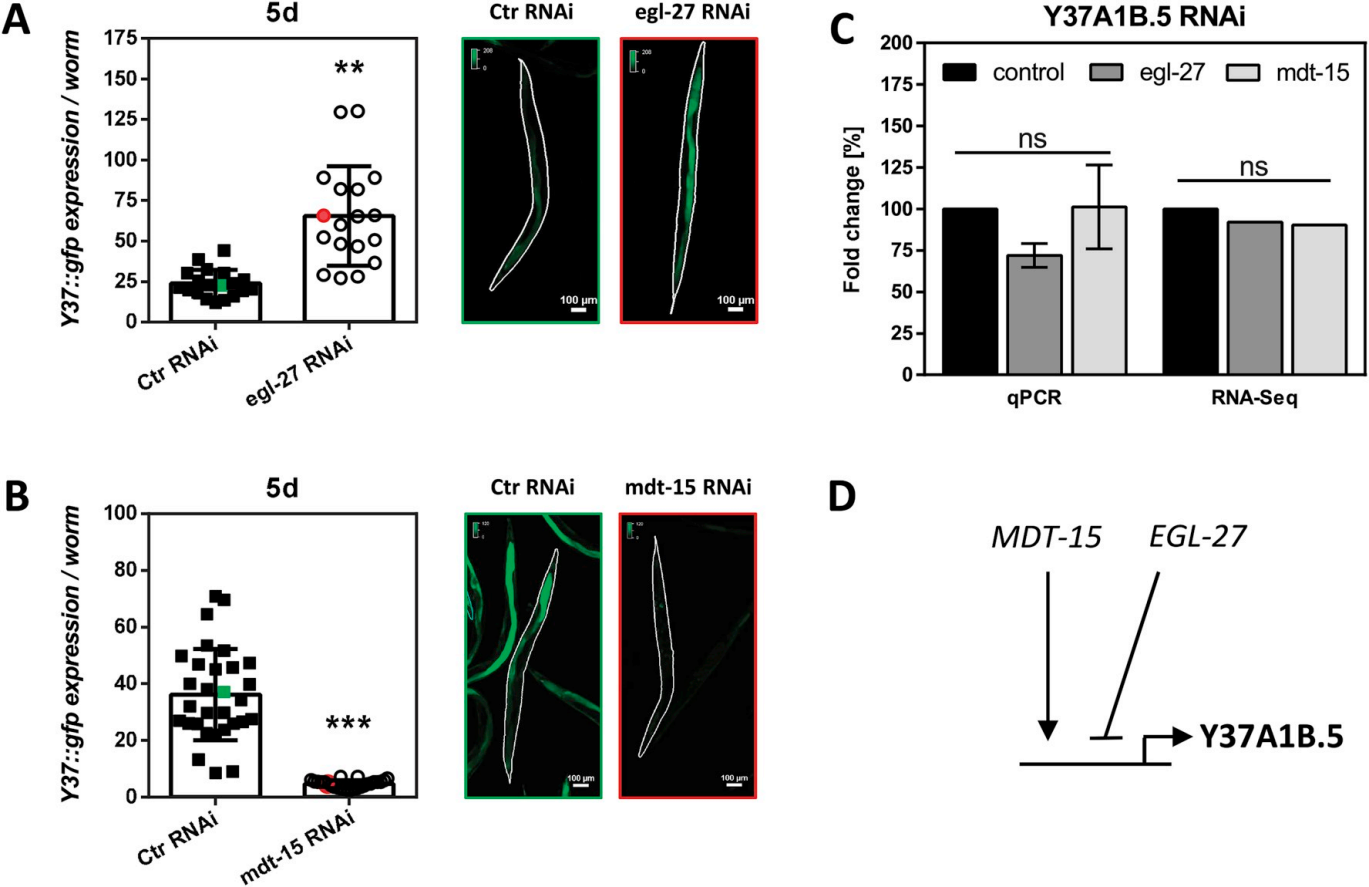
MDT-15 and EGL-27 modulate the expression of *Y37A1B.5*. **(A, B)** Young adult worms overexpressing *pY37A1B.5*::*Y37A1B.5*::*gfp* (see Fig. 3) were treated for 5 days, using control plasmids or (A) *egl-27*-specific or (B) *mdt-15*-specific RNAi plasmids. Each data point represents Y37A1B.5::GFP expression/worm in arbitrary units for one individual worm. Right panels show examples of worms evaluated, representing worms exposed to control (green box) or target-specific RNAi plasmids (red box). Dashed white lines indicate shapes of worms. **(C)** Expression of *egl-27* and *mdt-15* at day 5 of *Y37A1B.5* knockdown, as evaluated by qRT-PCR and RNA-Seq, respectively. **(D)** Schematic representation of MDT-15 and EGL27 regulation of *Y37A1B.5* expression.

RNAi of *egl-27* resulted in upregulation of Y37A1B.5::GFP production, implying that EGL-27 is a negative regulator of *Y37A1B.5* expression (Fig. 8A). This is in line with the notion that downregulation of *Y37A1B.5* enhances stress resistance and lifespan of *C. elegans*. *Y37A1B.5* was previously identified as an EGL-27 target gene whose expression levels are lower in *egl-27*-deficient cells, and that is upregulated by heat stress only in the presence of EGL-27 [40]. These data contradict our findings on the negative effect of EGL-27 on Y37A1B.5 levels. A possible reason for these differences is that we analyzed for expression at the protein level whereas the data by Xu and Kim [40] refer to analyses at the mRNA level. Another difference is that we used RNAi-mediated downregulation of *egl-27*, whereas Xu and Kim had *egl-27* expression completely inactivated.

Downregulation of *mdt-15* using RNAi causes a robust downregulation of Y37A1B.5 (Fig. 8B), suggesting it is directly involved in its transcriptional control. The knockdown of *mdt-15* would, according to our data, imitate the RNAi-induced downregulation of *Y37A1B.5,* eliciting an enhanced stress resistance (Fig. 4) and extending lifespan (Fig. 2). Interestingly, MDT-15 appears to control the expression of lipid metabolism genes and the nematodal oxidative stress response by interacting with both NHR-49 and SKN-1 [38,41]. Knockdown of *Y37A1B.5*, however, did not affect *mdt-15* or *egl-*27 mRNA levels (Fig. 8C).

In summary, *Y37A1B.5* expression is negatively regulated by EGL-27 and stimulated by MDT-15 (Fig. 8D).

## 4 Conclusions

Y37A1B.5, a protein hitherto uncharacterized, is a *C. elegans* ortholog of SELENBP1 that allows for a better survival of nematodes exposed to toxic selenite concentrations, while it is dispensable for adult worms grown under standard laboratory conditions. In fact, knockdown of *Y37A1B.5* in young adult *C. elegans* even resulted in an increased lifespan and resistance against oxidative stress. These effects are associated with changes in the *C. elegans* transcriptome that suggest a contribution of sulfur metabolic processes and of fuel metabolism. Surprisingly, the transcription factors DAF-16 and NHR-49, which are prominent regulators of fuel metabolism and stress resistance in *C. elegans*, are not involved in the lifespan extension mediated by *Y37A1B.5* knockdown. However, we identify two novel regulators of *Y37A1B.5* expression, which is negatively regulated by EGL-27 and positively regulated by MDT-15.

Our findings on Y37A1B.5 conferring selenite resistance, and its downregulation contributing to oxidative stress resistance and lifespan extension are in line with observations on the human ortholog: SELENBP1 sequesters supplemental Se, and its downregulation in tumor cells decreases their sensitivity to cytotoxic effects of oxidative stressors such as H_2_O_2_ and paraquat [42]. On the other hand, expression of Y37A1B.5 can be advantageous for free-living nematodes, as they are widely spread geobionts and thus have to cope with large variations in the Se content of soils world-wide, ranging from 0.01 to 1200 mg Se/kg [2].
