# Supplementary material for: A Caenorhabditis elegans ortholog of human selenium-binding protein 1 is a pro-aging factor protecting against selenite toxicity
Source: Redox Biol. 2019 Sep 11;28:101323. doi: 10.1016/j.redox.2019.101323 (PMC6812014; doi:10.1016/j.redox.2019.101323)

A *Caenorhabditis elegans* ortholog of human selenium-binding protein 1 is a pro-aging factor protecting against selenite toxicity

Karl Köhnlein, Nadine Urban, David Guerrero-Gómez, Holger Steinbrenner, Pavel Urbánek, Josephine Priebs, Philipp Koch, Christoph Kaether, Antonio Miranda-Vizuete, Lars-Oliver Klotz

-- SUPPLEMENTARY FIGURES --

The following supplementary figures show survival curves of all individual experiments listed in Tables 2 and 4. Those supplementary figures shown also in any of the regular manuscript figures are boxed and indicated below. They are copied here for the sake of completely mirroring the data given in the above-referenced tables.

Fig. S1 Knockdown of *Y37A1B.5* (starting at young adult stage). For data underlying the figures, see Table 2. Figure S1A is also shown in Figure 2C.

Fig. S2 Knockdown of *Y37A1B.5* (starting from egg). For data, see Table 2.

Fig. S3 Knockdown of *Y37A1B.5* in *daf-16(mu86)*. For data, see Table 4. Figure S3A is also shown in Figure 7A.

Fig. S4 Knockdown of *Y37A1B.5* in *skn-1(zu135)*. For data, see Table 4.

Fig. S5 Knockdown of *Y37A1B.5* in *nhr-49(ok2165)*. For data, see Table 4. Figure S5B is also shown in Figure 7B.

Fig. S1 Knockdown of *Y37A1B.5* (starting at young adult stage)

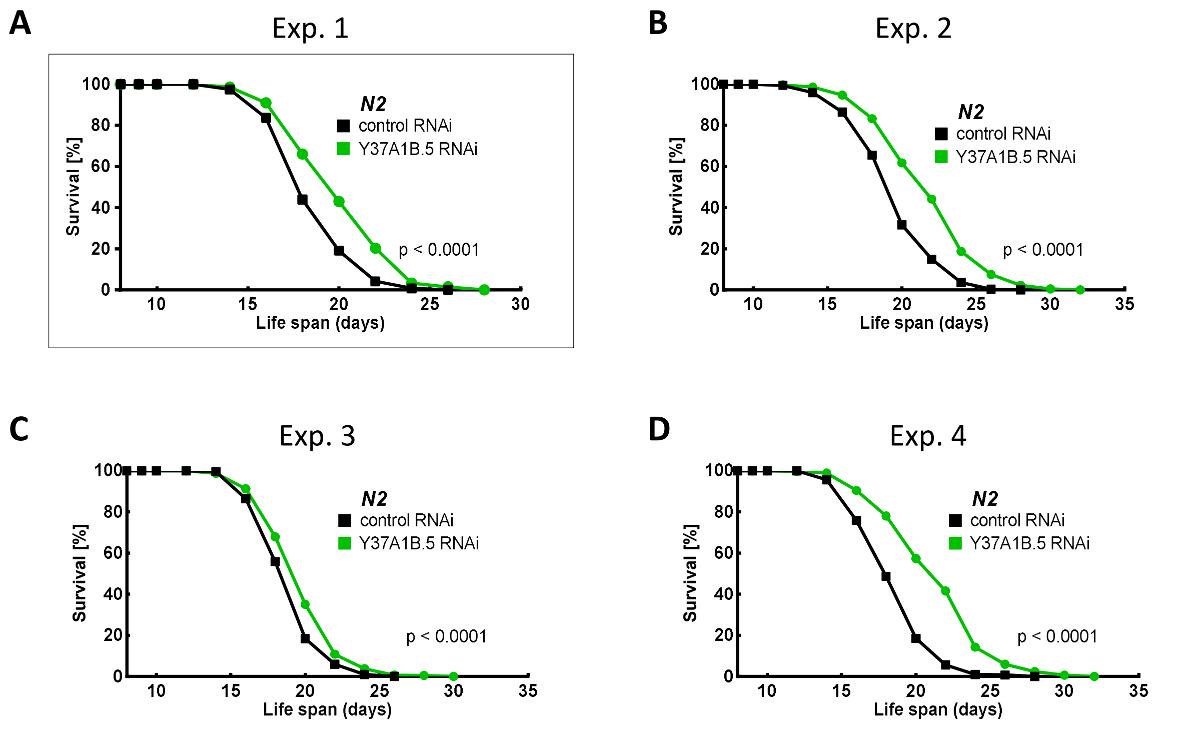

Fig. S2 Knockdown of *Y37A1B.5* (starting from egg)

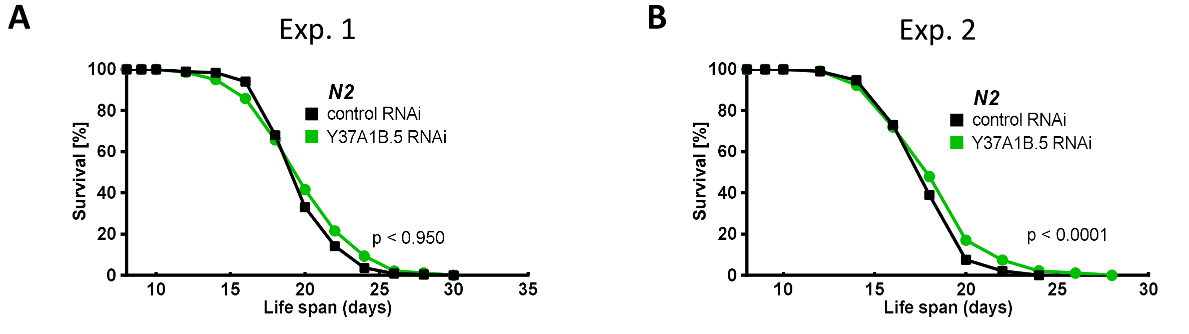

Fig. S3 Knockdown of *Y37A1B.5* in *daf-16(mu86)*

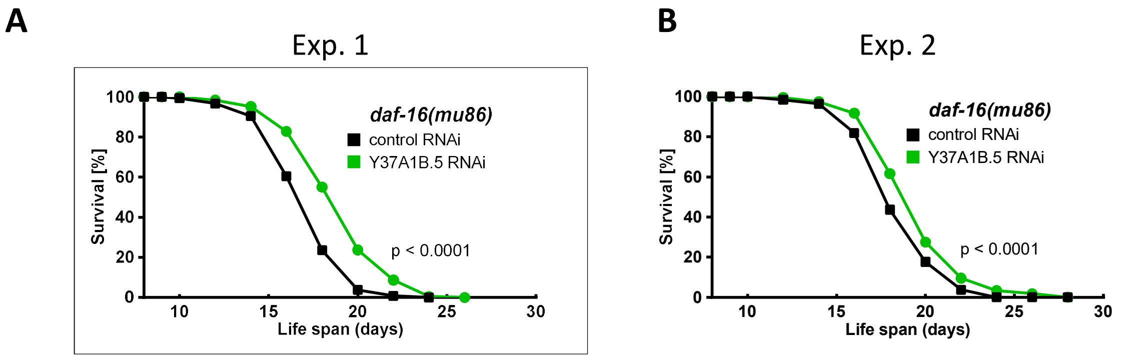

Fig. S4 Knockdown of *Y37A1B.5* in *skn-1(zu135)*

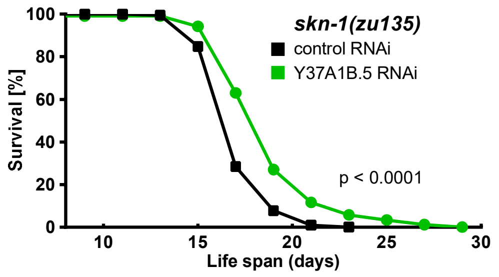

Fig. S5 Knockdown of *Y37A1B.5* in *nhr-49(ok2165)*

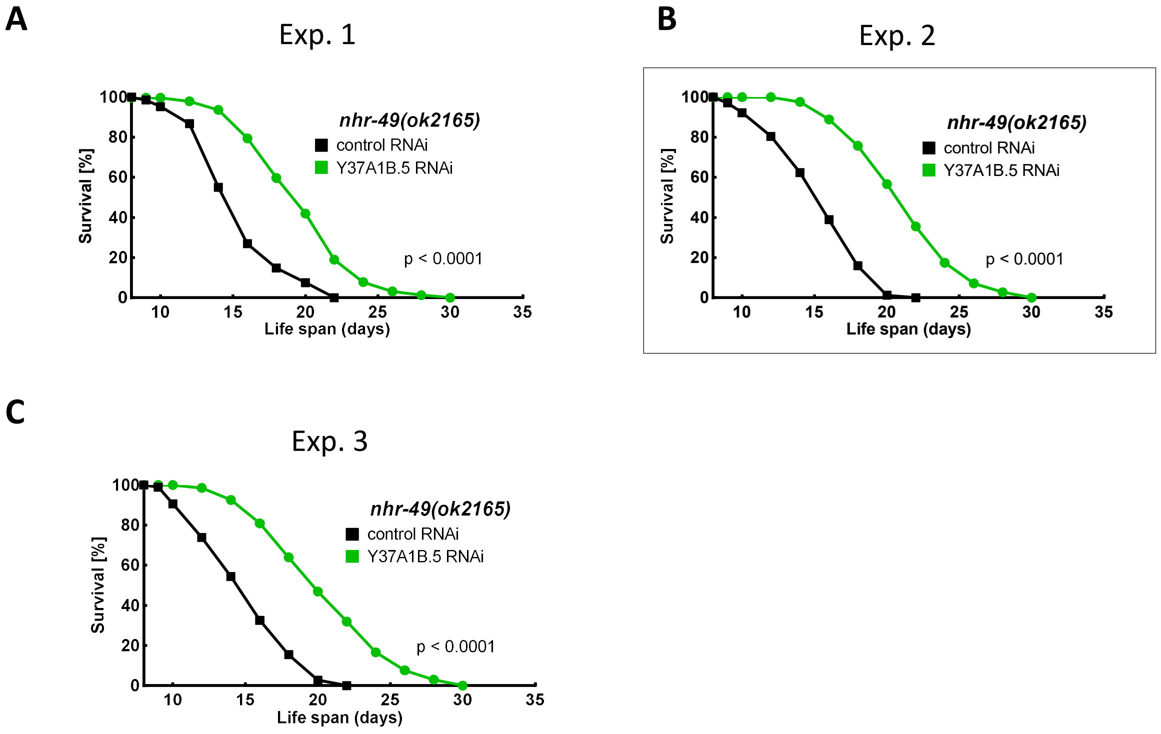

Supplement: Multimedia component 1 [file mmc1.pdf]
